# Ruptured Common Femoral Artery Aneurysm or Abdominal Aortic Aneurysm?

**DOI:** 10.1155/2013/306987

**Published:** 2013-12-09

**Authors:** Tetsuya Niino, Satoshi Unosawa, Haruka Kimura

**Affiliations:** Department of Cardiovascular Surgery, National Hospital Organization Disaster Medical Center, 3256 Midori-cho, Tachikawa, Tokyo 190-0014, Japan

## Abstract

We encountered a patient with a large retroperitoneal hematoma due to rupture of a common femoral artery aneurysm. A 77-year-old man was transferred to our hospital with left groin pain and shock. Computed tomography demonstrated a large retroperitoneal hematoma involving the left iliofemoral segment with extravasation of contrast into the left groin from a ruptured left common femoral artery aneurysm. The patient also had an abdominal aortic aneurysm. Reconstruction of the common femoral artery with a graft was performed successfully. The patient had an uneventful postoperative course and subsequently underwent Y-graft replacement of the abdominal aortic aneurysm.

## 1. Introduction

True arteriosclerotic aneurysms of the femoral artery are rare and these patients often also have aneurysms in the aorta or in another peripheral artery [1]. Rupture of a common femoral artery (CFA) aneurysm is even rarer. Here we report a case of rupture of a left CFA aneurysm associated with shock and complicated by an abdominal aortic aneurysm (AAA).

## 2. Case Report

**Figure 1:**
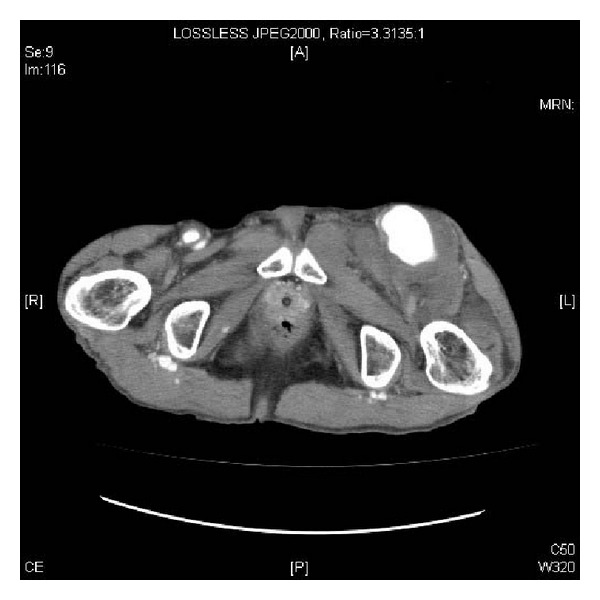
CT demonstrates a left femoral artery aneurysm, measuring 60 × 55 mm in size.

**Figure 2:**
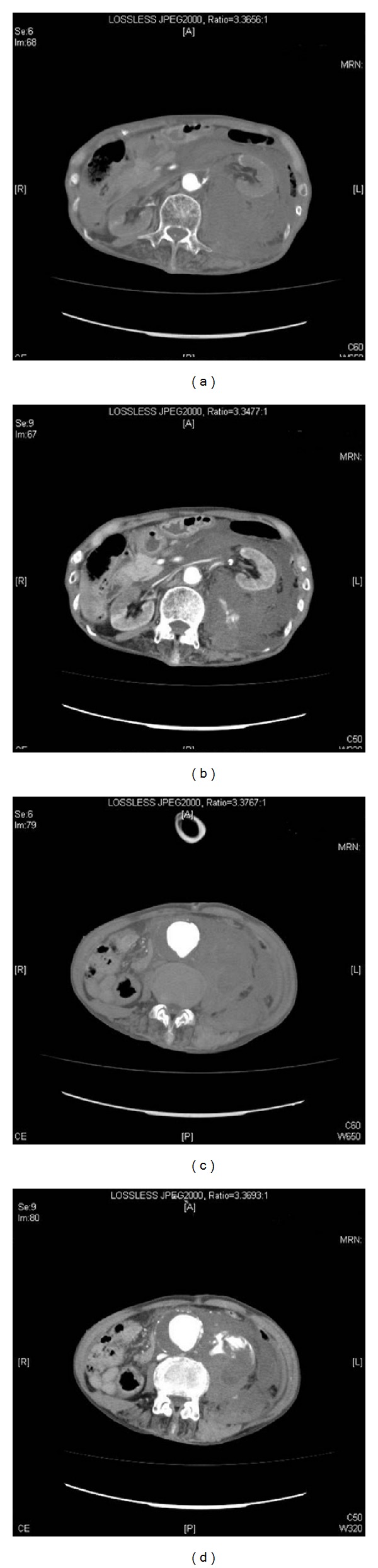
CT reveals a large retroperitoneal hematoma and abdominal aortic aneurysm. In the early phase, there is no contrast enhancement ((a) and (c)), but this is seen in the late phase ((b) and (d)).

**Figure 3:**
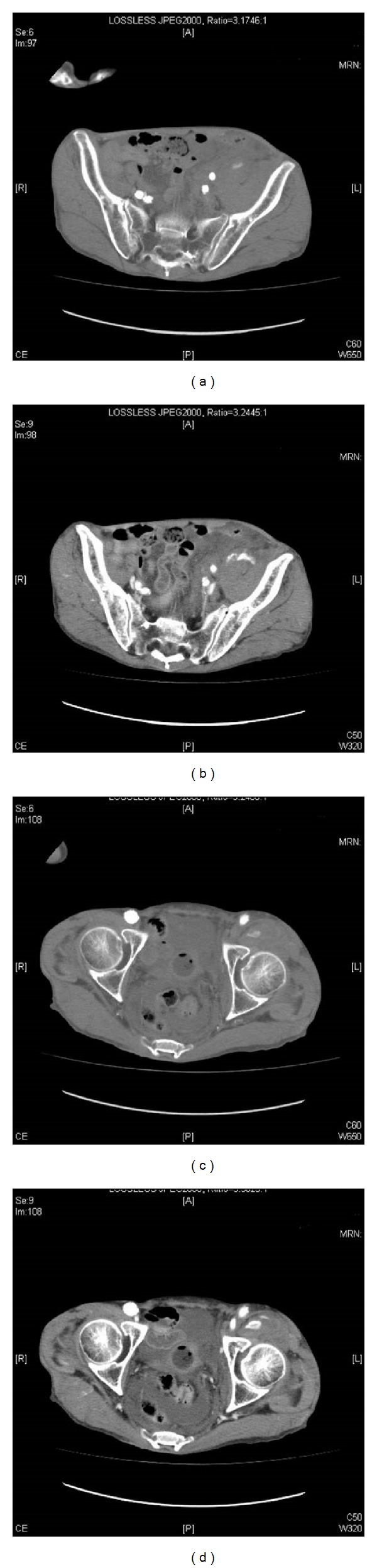
CT shows extravasation of contrast medium into the left groin from the left iliofemoral segment ((a) and (c)). In the late phase, extravasation and pooling of contrast medium is seen ((b) and (d)).

A 77-year-old man was transferred to our hospital with a 3-day history of left groin pain and subcutaneous hemorrhage. On arrival, he showed loss of consciousness and was in shock (blood pressure: 50/32 mmHg). He has a pulsating mass and hematoma in the left groin region. His hemoglobin was 4.0 mg/dL and his platelet count was 77,000. Blood gas analysis revealed severe metabolic acidosis (pH: 7.123) and an increased base excess (−14.7 mmol/L). Computed tomography (CT) showed the presence of a large retroperitoneal hematoma involving the left iliofemoral segment with extravasation of contrast medium into the left groin in the early phase of a ruptured left CFA aneurysm. There was also an infrarenal AAA (Figures 1, 2, and 3).

The patient was taken to the operating theater as soon as possible. Due to the patient's extremely unstable hemodynamic state, an oblique left inguinal incision was made rapidly to allow manual compression of the left external iliac artery. His hemodynamics became stable after manual compression was initiated. Then a separate longitudinal groin incision was made and the CFA was dissected. After the CFA was clamped, the aneurysm was opened to reveal severe atherosclerotic changes and a defect measuring about 2 cm in the posterior wall. Reconstruction of the common femoral artery was successfully performed with a 10 mm polytetrafluoroethylene (PTFE) graft.

The patient's postoperative course was uneventful. He was discharged and was later readmitted for elective Y-graft replacement of the AAA.

## 3. Discussion

True femoral artery aneurysms are caused by weakening of the arterial wall due to atherosclerosis and are predominantly found in elderly men, with the risk factors including hypertension and smoking [2]. In the present case, the patient had history of hypertension and smoking.

It has been reported that symptomatic femoral artery aneurysms or those larger than 2.5 cm in diameter should be treated in order to prevent limb threatening complications, such as rupture, thrombosis, or embolization [3]. In addition, the annual rupture rate of CFA aneurysms larger than 5 cm in diameter was reported to be 16% [4].

Femoral artery aneurysms are often bilateral and are clearly associated with the occurrence of other aneurysms, such as abdominal or thoracic aneurysms [5]. Ruptured AAA has been reported in patients who underwent initial femoral aneurysm repair and deferred aortic surgery [6]. We performed graft replacement of the AAA after six months to the present patient. It is important for the timing of aortic and femoral aneurysm surgery to be carefully evaluated in such cases.

In our patient, extravasation of contrast medium from the CFA aneurysm was detected in the early phase on CT, but we could not determine whether bleeding was from the CFA or AAA in the late phase. A benefit of dual-phase contrast CT for detection of leaks after endovascular repair and trauma has been reported [7, 8]. Similarly, dual-phase contrast-enhanced CT was useful for detecting the site of rupture in the present patient with multiple aneurysms.

In conclusion, we presented a case of ruptured CFA associated with AAA. A patient with a femoral artery aneurysm is likely to also have one or more other aneurysms and should undergo screening. Early and late phase contrast-enhanced CT scanning is useful for detecting the source of bleeding in patients with multiple aneurysms.
